# Pathological variants in genes associated with disorders of sex development and central causes of hypogonadism in a whole-genome reference panel of 8380 Japanese individuals

**DOI:** 10.1038/s41439-022-00213-w

**Published:** 2022-09-28

**Authors:** Naomi Shiga, Yumi Yamaguchi-Kabata, Saori Igeta, Jun Yasuda, Shu Tadaka, Takamichi Minato, Zen Watanabe, Junko Kanno, Gen Tamiya, Nobuo Fuse, Kengo Kinoshita, Shigeo Kure, Akiko Kondo, Masahito Tachibana, Masayuki Yamamoto, Nobuo Yaegashi, Junichi Sugawara

**Affiliations:** 1 Graduate School of Medicine, Tohoku University, 2-1, Seiryo-machi, Aoba-ku, Sendai, 980-8575 Japan; 2 Tohoku Medical Megabank Organization, Tohoku University, 2-1, Seiryo-machi, Aoba-ku, Sendai, 980-8573 Japan; 3 Miyagi Cancer Center Research Institute, 47-1, Nodayama, Medeshima-Shiode, Natori, 981-1293 Japan; 4 Statistical Genetics Team, RIKEN Center for Advanced intelligence Project, Nihonbashi 1-chome Mitui Building, 15th Floor, 1-4-1 Nihonbashi, Chuo-ku, Tokyo, 103-0027 Japan; 5 Graduate School of Information Sciences, Tohoku University, 6-3-09, Aza-aoba, Armaki, Aoba-ku, Sendai, 980-8579 Japan; 6 Institute of Development, Aging and Cancer, Tohoku University, 4-1 Seiryo-machi, Aoba-ku, Sendai, 980-8575 Japan; 7 Advanced Research Center for Innovations in Next-Generation Medicine, Tohoku University, 2-1 Seiryo-machi, Aoba-ku, Sendai, 980-8573 Japan

**Keywords:** Genetic variation, Medical genetics

## Abstract

Disorders of sex development (DSD) comprises a congenital condition in which chromosomal, gonadal, or anatomical sex development is atypical. In this study, we screened for pathogenic variants in 32 genes associated with DSDs and central causes of hypogonadism (CHG) in a whole-genome reference panel including 8380 Japanese individuals constructed by Tohoku Medical Megabank Organization. Candidate pathogenic (P) or likely pathogenic (LP) variants were extracted from the ClinVar, InterVar, and Human Gene Mutation databases. Ninety-one candidate pathological variants were found in 25 genes; 28 novel candidate variants were identified. Nearly 1 in 40 (either ClinVar or InterVar P or LP) to 157 (both ClinVar and InterVar P or LP) individuals were found to be carriers of recessive DSD and CHG alleles. In these data, genes implicated in gonadal dysfunction did not show loss-of-function variants, with a relatively high tendency of intolerance for haploinsufficiency based on pLI and Episcore, both of which can be used for estimating haploinsufficiency. We report the types and frequencies of causative variants for DSD and CHG in the general Japanese population. This study furthers our understanding of the genetic causes and helps to refine genetic counseling of DSD and CHG.

## Sexual development disorders: Variant prevalence in the Japanese population

Researchers in Japan have reported the frequency of mutations related to atypical sexual development in the Japanese population. A team led by Junichi Sugawara of Tohoku University, Sendai, analyzed whole-genome data collected by the Tohoku Medical Megabank Organization from 8380 healthy Japanese people. They screened the genomes for variations in 32 genes linked with central hypogonadism (CHG) and disorders of sex development (DSD), and used bioinformatics to predict the effect of the variants. This revealed 91 potentially pathogenic variants in 25 of the genes, including 28 new variants. The team also estimated the frequency of unaffected carriers of these variants. The analysis provides an overview of the prevalence of these variants in the general Japanese population and also offers valuable data for genetic diagnosis and counseling related to DSD and CHG.

## Introduction

Sexual differentiation proceeds under the control of the genetic program that governs the differentiation and development of an individual’s sexual phenotype through a sequential cascade of chromosomal, gonadal, and genital differentiation. Those with conditions that deviate from this program have historically been described as “intersex”. “hermaphrodite”, and “pseudohermaphrodite”. These discriminatory terms were replaced with the general “disorders of sex development (DSD)” in 2006^1,2^. DSD increases the risk of psychosocial problems, including anxiety, depression, and decreased quality of life, similar to what is seen in patients with chronic illnesses^3^. DSD includes 1) sex chromosome variations (sex chromosome DSD), 2) disorders of testis development and androgenization (46,XY DSD), and 3) disorders of ovary development and androgen excess (46,XX DSD)^2^. The clinical manifestations of DSD vary, and the variation in phenotypes reflects the diversity of DSD causes. Most DSD cases are apparent at birth or earlier because of ambiguities in the internal and/or external genitalia; the few remaining cases are diagnosed after puberty based on slow or atypical sexual maturation, such as amenorrhea, gonadal dysfunction, or infertility. DSD is rare in the general population, at 2:10,000^4,5^. Thyen et al. examined cases of ambiguous genitalia in the German rare-disease registry; DSD not diagnosed by infantile ambiguous genitalia was not included^5^. Therefore, the true incidence rate of DSD in the general population is unknown.

Recent technological advances in the characterization of genetic variations, such as next-generation sequencing (NGS), allow for the discovery of new variants in patients with DSD^6^. The development of a 30-gene NGS panel for DSD is reportedly useful for genetic diagnosis of DSD, as well as for genetic counseling and personalized patient treatment^7^. While genetic diagnosis has been reported for 43–60% of patients with DSD, the actual incidence rate is different from the proportions of potential patients with causative variants^8,9^.

Overall, knowledge of the true estimated frequency and variant types based on the frequency of carriers in the general population is limited. The frequency of DSD-related gene variants in the general population has appeared in reports^10,11^ investigating the frequency of carriers of the gene responsible for adrenocortical hyperplasia (*CYP21A2*) in up to 1000 individuals. However, there are no reports on the types and frequency of DSD-related gene variants in the general Japanese population.

In general, identifying pathogenic variants of DSD in the general Japanese population will facilitate precise diagnosis and genetic counseling for DSD in gynecologic and pediatric endocrine practice, enhancing patient quality of life. We aimed to study not only DSD-related genes but also genes related to central causes of hypogonadism (CHG) to comprehensively investigate variants of genes related to gonadal development.

The Tohoku University Medical Megabank Organization (ToMMo) and Iwate Medical University have initiated genome cohort studies using an integrative biobank that integrates subjects’ medical and genomic data^12^. One aim is to develop precise whole-genome reference panels for the Japanese population by providing information on genomic variants^13–15^. ToMMo released 8.3KJPN, a panel of short variants of 8380 Japanese individuals precisely identified using whole-genome sequencing (https://jMorp.megabank.tohoku.ac.jp/202102/). It consists of ~86 Mb autosomal genomic variants (both single-nucleotide and short indels <50 bp), including abundant low-frequency variants, and can be used to explore the prevalence of DSD and CHG with autosomal recessive inheritance in the Japanese population.

In this study, we focused on DSD- and CHG-related genes with recessive inheritance and the androgen receptor gene (*AR*), deleterious mutation of which causes X-linked recessive DSD. ToMMo subjects are suitable for this analysis because they were screened as healthy Japanese individuals. We investigated the types and frequencies of causative variants in ToMMo subjects and estimated carrier frequencies with causative variants. In addition, we examined the paucity of loss-of-function (LOF) variants in a subset of 32 selected genes.

## Materials and methods

### DEGs and CHG-related genes

We chose genes based on Consensus Statement on Management of Intersex Disorders proposed by The Lawson Wilkins Pediatric Endocrine Society and European Society for Pediatric Endocrinology in 2016^1^ as well as current studies on DSD and CHG-related genes and candidate genes^1,9,16,17^. We searched for descriptions of DSD- and CHG-related genes in PubMed and in clinical variant databases such as ClinVar, HGMD, and OMIM. We focused on 31 genes with autosomal recessive inheritance as well as X-linked *AR*. We excluded known DSD-causing autosomal-dominant variants because they are rare and it is, therefore, difficult to predict precise carrier frequencies. Table 1 shows the 32 selected genes, the disease associated with each, their MIM numbers, and their phenotype MIM numbers.

**Table 1:** DSD- and CHG-related genes chosen for study.

Disease		Gene	MIM#	Phenotype MIM#
Gonadal dysfunction (GD)				
	46, XY DSD, complete gonadal dysgenesis (CGD) (Sywer syndrome)	*CBX2*	602770	613080
	46, XY DSD, partial gonadal dysgenesis (PGD), CGD	*DHH*	605423	233420
	46, XY DSD, sudden infant death with dysgenesis of the testes (SIDDT)	*TSPYL1*	604714	608800
	46, XX ovo-testicular DSD with palmoplantar hyperkeratosis	*RSPO1*	609595	610644
	46, XX testicular DSD with dysgenesis of kidney, adrenals, and lungs (SERKAL syndrome)	*WNT4*	603490	611812
Disorders in hormone synthesis or action (HSA)				
	46, XX DSD, Congenital adrenal hyperplasia (CAH)	*CYP11B1*	610613	202010
		*HSD3B2*	613890	201810
		*CYP21A2*	613815	201910
	46, XY DSD, Congenital adrenal hyperplasia (CAH)	*STAR*	600617	201710
		*CYP17A1*	609300	202110
	46, XY DSD with adrenal insufficiency. CAH	*CYP11A1*	118485	613743
	46, XY DSD, Persistent Mullerian ducts syndrome, type I	*AMH*	600957	261550
	46, XY DSD, Persistent Mullerian ducts syndrome, type II	*AMHR2*	600956	261550
	46, XX DSD, Aromatase deficiency	*CYP19A1*	107910	613546
	46, XY DSD, 17-b hydroxysteroid dehydrogenase 3 deficiency	*HSD17B3*	605573	264300
	46, XY DSD, Leydig cell hypoplasia Luteinizing hormone resistance in females	*LHCGR*	152790	238320
	46, XY DSD, 5-a reductase deficiency	*SRD5A2*	607306	264600
	46, XY DSD	*AKR1C4*	600451	614279
		*AKR1C2*	600450	614279
	46, XX DSD, 46, XY DSD, Cytochrome P450 oxidoreductase deficiency (PORD)	*POR*	124015	201750
	46, XY DSD with Methemoglobinemia	*CYB5A*	613218	250790
	46, XX DSD (Perrault syndrome 1)	*HSD17B4*	601860	233400
	46, XY DSD, Smith-Lemli-Opitz syndrome	*DHCR7*	602858	270400
	46, XY DSD, Androgen insensitivity/Hypospadias	*AR*	313700	300068/300633
Central causes of hypogonadism (CHG)				
	Hypogonadotropic hypogonadism	*GNRHR*	138850	146110
		*GNRH1*	152760	614841
		*TAC3*	162330	614839
	Leptin deficiency	*LEP*	164160	614962
	Pituitary hormone deficiency	*PROP1*	601538	262600
		*LHX3*	600577	221750
	Ovarian dysgenesis	*FSHR*	136435	233300
	Bardet-Biedl syndrome	*BBS9*	607968	615986

For 32 DSD- and CHG-related genes, MIM#, Phenotype MIM# are presented.

### Data source and variant annotation

The whole-genome reference Panel 8.3KJPN^18^ was constructed from whole-genome sequences of 8380 healthy Japanese individuals from ToMMo and Iwate Medical University Tohoku Medical Megabank (IMM). Written informed consent for participation and publication was obtained from each IMM participant and other prospective cohorts in Japan (JPHC-NEXT, J-MICC, Nagahama, and Nagasaki cohorts). This study was approved by the Ethics Committee of Tohoku Medical Megabank Organization (ToMMo) at Tohoku University (authorization number: 2018-4-043).

The original database on allele frequencies for 8.3KJPN is available from the portal site, Japanese Multi Omics Reference Panel (http://jMorp.megabank.tohoku.ac.jp/)^18^, and downloadable as VCF files. Multiallelic variants were split into individual alleles using bcftools^19^ by using the “norm –m – (minus)” option, and their allelic frequency was assigned accordingly. In the case of *AR*, we used tommo-8.3kjpn-20200831-af_snvall-chrX_PAR2.vcf.gz and tommo-8.3kjpn-20200831-af_indelall-chrX_PAR2.vcf.gz to obtain minor allele frequencies (MAF) of the variants.

Multiple analyses were conducted using the GRCh37/hg19 genomic coordinates. Variants in 8.3KJPN were annotated using the ClinVar July 2020 version^20^, the professional version of Human Gene Mutation Database (HGMD: February 2020)^21^, and InterVar version 2.0.2^22^ including Annovar^23^. InterVar assesses the pathogenicity of gene variants using 28 criteria based on the 2015 guidelines of American College of Medical Genetics and Genomics and Association for Molecular Pathology^24^ with 18 (PVS1, PS1, PS4, PM1, PM2, PM4, PM5, PP2, PP3, PP5, BA1, BS1, BS2, BP1, BP3, BP4, BP6, and BP7) of the 28 criteria implemented for automatic interpretation. Combined files of variant annotations and primary interpretation (ClinVar, HGMD, InterVar including Annovar) were created for each causative gene; genotype frequencies and original multiple alleles were added.

### Detection of pathogenic variants

**Fig. 1:**
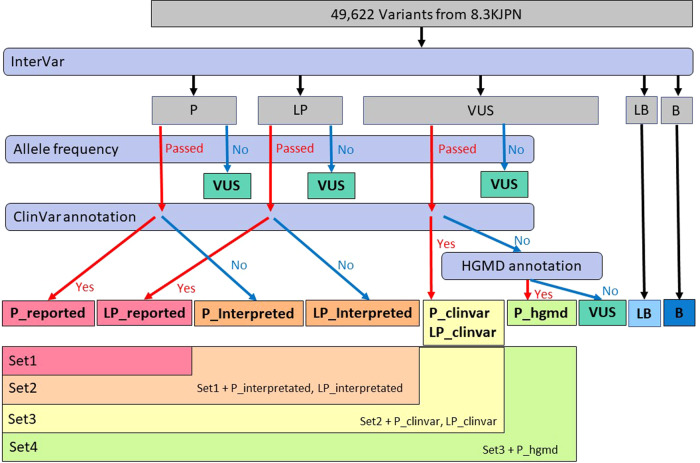
Schematic diagram of candidate DSD- and CHG-causing variant classification. Filtering steps are indicated as rounded rectangles. P, pathogenic; LP, likely pathogenic; VUS, variants with uncertain significance; B, benign; and LB, likely benign.

Using the annotation output for 8.3KJPN, variants in 32 genes were selected for analysis by including regions 1 kb upstream and downstream. We classified the genomic variants and selected pathogenic variants using multiple criteria, as described previously^25^, with minor modifications (Fig. 1). The 8.3KJPN database includes variants with Variant Quality Score Recalibration (VQSR) scores (VQSRTrancheINDEL99.00to99.90 and VQSRTrancheSNP99.50to99.60). We removed variants with VQSR scores, and the remaining variants (“PASS”ed) were then annotated. We analyzed variants using InterVar and sorted them into five classes: pathogenic (P), likely pathogenic (LP), variant of uncertain significance (VUS), likely benign (LB), and benign (B). We further classified P and LP variants into reported or predicted using HGMD disease-causing (DM) variants and ClinVar variants (pathogenic or likely pathogenic) filtering using a threshold of MAF of <0.03. Detection of pathogenic variants and their estimated carrier frequencies was performed using four different inclusion criteria originating from Yamaguchi-Kabata et al.^26^. The following is an overview of the four different inclusion criteria from Sets 1 to 4. Set 1 is the strictest selection; Set 4 includes a broad range of possibilities. Set 1 was defined as the most conservative pathogenic variants, with class P and LP corresponding to ClinVar. Set 2 was defined as all class P and LP variants. Set 3 was defined as Set 2 together with the ClinVar variants in class VUS. Set 4 was defined as Set 3 together with the HGMD variants in class VUS.

The 8.3KJPN database includes whole-genome data generated using five different platforms (Illumina_HiSeq_2500 162PE, 27.2x; Illumina_HiSeq_2500 259PE, 21.3x; Illumina_HiSeqX_Five 150PE, 53.1x; Illumina_NovaSeq_6000 150PE, 30.1x; and MGI_DNBSeq_G400 150PE, 30.1x: Mapping quality >20); three of these (HiSeq 162PE, HiSeq 259PE, and NovaSeq 150PE) have relatively large amounts of sample data. 8.3KJPN variants were annotated considering possible platform bias based on *p* values (threshold: *p* < 0.001) for the MAFs from the three platforms using Fisher’s exact test.

### Classification of individual variant status

Using individual genotype data, we manually inspected homozygous genotypes and individuals with multiple pathogenic variants in a single gene. In addition, we investigated mRNA isoforms from the annotations for LOF variants (stop-gain, splicing, and frameshift indels) using the images of genome coordinates obtained from NCBI (genomic regions, transcripts, and products).

### Estimation of population frequencies of risk alleles and expected carriers

When there are *n* pathogenic variant sites in a causative gene, the sum of the MAF of *n* pathogenic alleles is defined as the estimated population frequency of pathogenic alleles of that gene (Q). We used Hardy–Weinberg equilibrium to estimate carrier frequencies; estimated frequencies of heterozygosity were calculated using 2 × (1−*Q*) × *Q*. We estimated the expected frequencies of individuals having pathogenic variants as a proportion of homozygotes or compound heterozygotes of pathogenic variants as *Q*^2^. In the case of *AR*, we calculated the probability as Q/3 because it is an X-linked gene.

### Tolerance of haploinsufficiency

The two scores pLI^27^ and Episcore^28^ were evaluated for the data provided in the supplementary results and processed using Linux shell commands (grep –f) to extract genes of interest. The scores for categories of DSD-causative genes were compared using Student’s *t* test.

## Availability of data and material

Data on the variants and number of genotypes are freely available from jMorp (https://jMorp.megabank.tohoku.ac.jp/202102/). The data used are individual genomic information; as such, they are private, and it would be possible to identify individuals while using them. Therefore, it is necessary to obtain approval for data access from the TMM prospective cohort project; specifically, users should obtain approval from the sample and data access committee of the TMM Biobank. Upon applying to this committee, Group of Materials and Information Management (dist@megabank.tohoku.ac.jp) in TMM at Tohoku University supports the procedures for data transfer.

## Results

### Categories of DSD- and CHG-related genes

Genes were classified into three groups^2^ (Table 1): disorders of gonadal development (GD), disorders in hormone synthesis or action (HSA), and CHG. GD genes play important roles in the development of gonads during embryogenesis; however, the molecular pathways that cause DSD are not fully characterized for most of these genes. Many HSA genes encode enzymes essential for the production of androgens, are critical regulators of androgen, and the androgen receptor in 46,XY males. In the case of 46,XX females, defects in genes encoding enzymes related to steroid biosynthesis lead to androgen excess and a masculinizing DSD phenotype. The CHG category primarily involves genes encoding proteins associated with hypogonadotropic hypogonadism and central pituitary hormone defects.

### Overview of potential pathogenic variants

We detected 49,622 genomic variants of the 32 DSD-causative genes in 8.3KJPN but found no discordance between genetic sex and self-reported sex. The variants in these 32 genes were classified (Fig. 1). First, genomic variants were classified as pathogenic (P; *n* = 13), likely pathogenic (LP; *n* = 29), variants of uncertain significance (VUS; *n* = 43,856), likely benign (LB; *n* = 445), and benign (B; *n* = 5279) based on annotation and interpretation using InterVar^22^. Second, possible pathogenic variants, including VUS, were filtered based on MAF < 0.03; all P and LP variants interpreted using InterVar passed this filtering. The P or LP variants included in both InterVar and ClinVar were allocated into Set 1, with P or LP variants included only in InterVar in Set 2. The variants annotated as P or LP in ClinVar but as VUS in InterVar were included in Set 3. Finally, HGMD DMs among the remaining variants were grouped in Set 4. The P or LP variants in InterVar were classified as P or LP_reported (Set 1; *n* = 14) and P or LP_interpreted (Set 2; *n* = 42) using annotations based on ClinVar significance. Among the VUSs interpreted using InterVar, annotations matching the classification criteria of ClinVar and HGMD were grouped as P and LP_ClinVar (Set 3; *n* = 57) and P_hgmd (Set 4; *n* = 91), respectively. The total number of candidate pathogenic variants included in Set 1–4 was 91 for the 25 genes. The 28 candidate pathogenic variants remaining after subtracting the candidate pathogenic variants in Set 1 from Set 2 have not been previously reported; they are novel variant candidates interpreted as having pathological importance in InterVar.

Ten individuals carried one pathogenic variant for two different DSD-causative genes. One individual showed two different potential *CYP21A2* gene pathogenic variants. One candidate SNV in the *POR* gene, c.G1738C:p. Glu580Gln, was homozygous in one individual; however, the variant is P_hgmd, and the pathogenicity of P_hgmd variants tends to be overestimated^29,30^. This individual has been included in other prospective cohorts studied in collaboration with the Tohoku Medical Megabank Project; however, except for sex, a detailed phenotype was not available.

Candidate pathogenic variants were detected for 25 of the 32 total genes but not for the remaining eight (*DHH, RSPO1, TSPYL1, AKR1C4, AKR1C2, CYB5A, GNRH1*, and *TAC3*) (Table 2). Four genes (*AMH, AMHR2, AR*, and *FSHR*) showed only P_hgmd variants. Many of these genes encode hormones, hormone receptors, or signaling molecules (*DHH, RSPO1, GNRH1, AMH, AMHR2, AR*, and *FSHR*). The cumulative allele frequencies and detailed information for the candidate pathogenic variants are summarized in Supplementary Tables 1 and 2, respectively.

**Table 2:** Numbers of candidate pathogenic variants of 32 genes for DSD and CHG.

	Set 1^a^	Set 2^a^	Set 3^a^	Set 4^a^
Gene	No. Var	No. Var	No. Var	No. Var
*CBX2*	0	2	2	2
*DHH*	0	0	0	0
*RSPO1*	0	0	0	0
*TSPYL1*	0	0	0	0
*WNT4*	0	1	1	1
*CYP11B1*	0	1	1	1
*CYP17A1*	1	1	5	6
*HSD3B2*	0	1	2	5
*STAR*	4	4	4	5
*CYP21A2*	0	0	1	2
*AMH*	0	0	0	5
*AMHR2*	0	0	0	3
*CYP19A1*	0	3	3	5
*HSD17B3*	2	8	8	9
*LHCGR*	0	1	1	1
*SRD5A2*	0	0	6	8
*AKR1C4*	0	0	0	0
*AKR1C2*	0	0	0	0
*POR*	3	6	7	10
*CYP11A1*	0	1	1	1
*CYB5A*	0	0	0	0
*HSD17B4*	0	1	1	3
*DHCR7*	0	2	3	9
*AR*	0	0	0	3
*GNRHR*	1	3	4	4
*GNRH1*	0	0	0	0
*TAC3*	0	0	0	0
*LEP*	1	1	1	1
*PROP1*	1	1	1	1
*LHX3*	1	1	1	1
*FSHR*	0	0	0	1
*BBS9*	0	4	4	4

### Potential disease-causing variants in GD genes

**Fig. 2:**
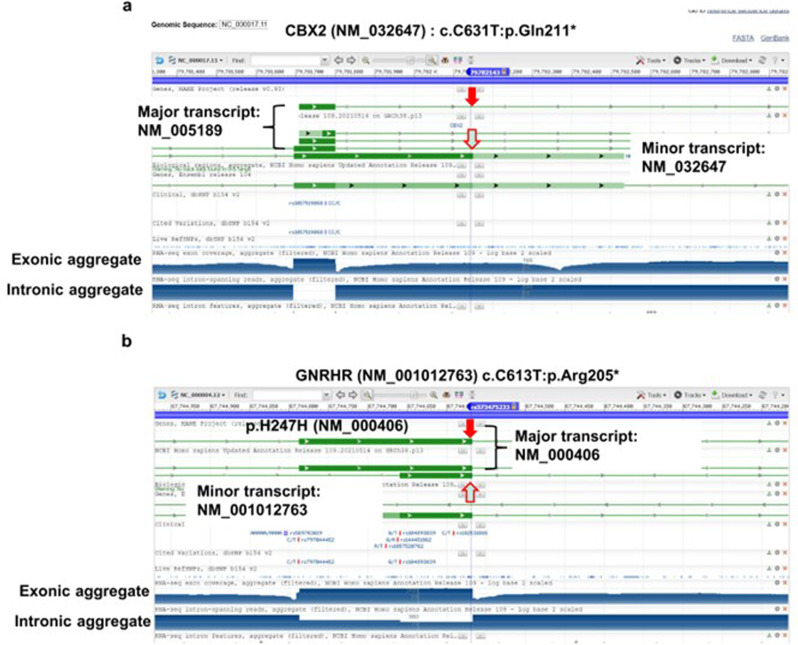
Candidate variants with isoform-specific annotations. NCBI website data for CBX2 (NM_032647): c.C631T:p. Gln211* (**a**) and GNRHR (NM_001012763) c.C613T:p. Arg205* (**b**). Red and orange arrows indicate the corresponding variant positions for major and minor transcripts, respectively. At the bottom of each panel, the exonic and intronic aggregates from RNA-seq data are indicated.

Three of five genes, *DHH, TSPYL1,* and *RSPO1*, in the GD category had no candidate disease-causing variants; the other two genes, *CBX2* and *WNT4*, had three variants classified as candidate disease-causing variants. No Set 1 or Set 3 variants were found in the genes of this category. Among the three potential disease-causing variants, only one allele, *CBX2* p. Gln211* (chr 17:g.77755943C>T) was annotated as an LOF variant in InterVar but not in ClinVar or HGMD (Supplementary Table 2). This functional annotation was based on the transcript NM_032647, which encodes a shorter isoform of *CBX2*. This variant results in a stop codon just before the termination codon of the short open-reading-frame isoform (Fig. 2a); Gln211 is the C-terminal amino acid of the short isoform of *CBX2*. This genomic variant is annotated as an intronic variant of the longer *CBX2* isoform (NM_005189). Considering that the major isoform of *CBX2* is NM_005189, based on aggregated RNA-seq data (see bottom of Fig. 2a), the variant chr17:g.77755943C>T should be annotated as a VUS after removing parameter PVS1 from the InterVar annotation data. The other six are nonsynonymous variants; one, *WNT4*: p. Phe315Ser, is annotated as VUS in ClinVar.

### Candidate disease-causing variants in HSA genes

This category includes 19 genes, many of which encode enzymes related to steroid metabolism. Fifty nonsynonymous variants were identified, with 30 belonging to Set 4 (P_hgmd only). Twenty-four candidate LOF variants were identified in this category. *CYP21A2* c.293-13C>G was found to be most common (8.3KJPN MAF = 0.00256) among known pathogenic DSD-causing variants. This variant is rs6467, and there is a much more frequent C>T variant (8.3KJPN MAF = 0.6658) at the same position. More than half of the candidate LOF variants (14) were classified as Set 2 or Set 4, indicating these 14 to be newly identified as P or LP by InterVar. Among them, the *CYP19A1* variant chr15: g.51630691C>T is ambiguous with respect to classification. It is annotated as a splice variant in InterVar and classified as an LP. However, based on refSeq data, the change occurs in the intergenic region of the major transcript; therefore, this variant should be interpreted as VUS. We found six potentially pathogenic variants in the *CYP17A1* gene in 8.3KJPN (Table 2 and Supplementary Table 3). We previously investigated this gene in a case of unambiguous female genitalia with 46,XY and identified two pathogenic mutations^31^, p. Phe53del and p. His373Leu, both of which were identified in 8.3KJPN. In the case of *AR*, we found no ClinVar, InterVar P, or LP variants; however, we identified three P_hgmd variants (Table 2 and Supplementary Table 2). None of the three *AR* variants in 8.3KJPN correspond to those identified in our previous study on androgen-insensitive syndrome cases^32^.

### Candidate disease-causing variants in CHG genes

This category consists of eight genes, many of which encode hormones or hormone receptors. We detected five nonsynonymous variants in this category, three of which are P or LP in ClinVar. Seven variants were considered to be LOF, with only one (*LHX3* c.G687A:p. Trp229*) being ambiguous: either P or LP in ClinVar. In the case of two stop-gain variants in the *GNRHR* gene, the annotations in InterVar are based on two different transcript isoforms. For rs373475233, the variant is annotated as c. C613T: p. Arg205* (NM_001012763). However, the transcript NM_001012763 is a minor transcript based on RNA-seq aggregate data from NCBI (Fig. 2b). In contrast, the *GNRHR* c.G618A:p. Typ206* (NM_000406) affects a major transcript of NM_000406; therefore, it might produce stop-gain transcripts.

### DSD carrier frequencies

After removing three ambiguous variants (*CBX2*: p. Gln211*, *GNRHR*: p. Arg205*, and *CYP19A1*: chr15:g.51630691C>T), the total MAFs of Set 1, Set 2, Set 3, and Set 4 were 0.00322, 0.00668, 0.01248, and 0.02997, respectively. Therefore, nearly one in 157 (Set 1), one in 73 (Set 2), one in 40 (Set 3), and one in 16 (Set 4) individuals were carriers of the gene alleles investigated in this study.

The individual distribution of the possible pathogenic variants of the causative genes in 8.3KJPN was examined to determine the compound heterozygosity or homozygosity of two pathogenic variants in causative genes in the case of recessive inheritance form. The probabilities of biallelic inactivation (BI) of the causative genes were calculated based on refined annotation data (Table 3). In the case of Set 1 (both ClinVar and InterVar annotated P or LP), most BI was HSA, and the total probability of Set 1 BI was one in 211 thousand births. Set 2 (InterVar P or LP) BI was possible for all categories, with a probability of 1 in 118 thousand births, nearly twice that for Set 1 BI. In the case of Set 3 BI (either ClinVar or InterVar annotated P or LP), only the HSA category showed a large increase from that of Set 2 BI (2.37-fold) relative to that of GD (no increase) and CHG (1.14-fold increase). This indicated that discrepancies between ClinVar and InterVar annotations occur more frequently in the HSA category. The BI probability for Set 3 was 1 in 50.7 thousand births. In the case of Set 4 (P or LP or DM in HGMD), HSA exhibited a more than sevenfold increase, and the Set 4 BI probability (without *AR* pathogenic variants) was 1 in 6787 births, suggesting false positives among the HGMD DM variants. In the case of *AR*, as there were only three HGMD DM mutants, the calculated probability was 0.000436 (1 in 2293 male births).

**Table 3:** Estimated proportions of homozygotes among pathogenic variants.

Category	Set 1	Set 2	Set 3	Set 4
GD	0	1.86E-07	1.86E-07	1.86E-07
HSA	4.71E-06	8.13E-06	1.93E-05	1.46E-04
AR^a^	0	0	0	4.37.E-04
CHG	1.44E-08	1.72E-07	1.97E-07	2.00E-07
Total^b^	4.73E-06	8.49E-06	1.97E-05	1.47E-04

^a^Hemizygous for male.

^b^AR is not included.

### Difficulty in variant calls for *CYP21A2*

Biallelic LOF variants in the *CYP21A2* gene are a major cause of 21-hydroxylase deficiency. Their prevalence is one in 15 to 18 thousand births in Japan^33,34^. Two candidate pathogenic variants were identified (Supplementary Table 3): c.293-13C>G (MAF = 0.00256) and p. Ala392Thr (MAF = 0.00584). The former belongs to Set 3 (ClinVar P or LP); it was homozygous in one in 152 thousand births. The latter belongs to Set 4 (only HGMD DM), being homozygous in one in 29 thousand births. The p. Ala392Thr allele is annotated as “Conflicting interpretations of pathogenicity” in the ClinVar database. If it is not a disease-causing variant, there would be only one other potential pathogenic variant for *CYP21A2;* hence, the expected incidence rate of BI (1/152,000) would be far too low relative to the observed ratio (1/18,000). Furthermore, we found nine ClinVar pathogenic variants in *CYP21A2* in the 8.3KJPN jMorp database (Supplementary Table 2). These were filtered out because they exhibited marginal quality in VQSR scoring (see methods).

### Distribution of LOF variants among causative categories and relationship to haploinsufficiency intolerance

The distribution of LOF variants among the three categories of DSD- and CHG-related genes is summarized in Supplementary Table 4. In the GD category, only one variant (CBX2 p. Glu211*) was annotated as LOF at initial classification, but a more detailed examination revealed that the annotation was ambiguous. Therefore, there were no definite LOF variants in the GD category; however, multiple LOF variants were identified in the other two categories (Supplementary Table 4). The paucity of GD LOF variants suggests that the genes in this category might be intolerant to hemizygous expression, suggesting haploinsufficiency.

To address this issue, two different parameters for haploinsufficiency tolerance were utilized: pLI and Episcore. The former stands for the probability of being loss-of-function intolerant and is based on the extent of depletion of LOF variants in the gnomAD project of more than 150 thousand exome sequencing samples^27^. Possible SNVs in genes were calculated using observed variant spectra with one flanking base (96 patterns) of the whole genome as prior probability; stop gains, splicing variants, and frameshifts were considered LOF variants. If the observed LOF variants in a gene are significantly lower than expected, the pLI would be high. The Episcore was developed to predict HIS using epigenomic data^28^. The hypothesis is that transcription of haploinsufficient genes is tightly regulated by epigenetic modifications and transcription factors. Machine learning was applied to epigenomic datasets of known haploinsufficient genes to calculate EpiScore (Supplementary Table 5).

**Fig. 3:**
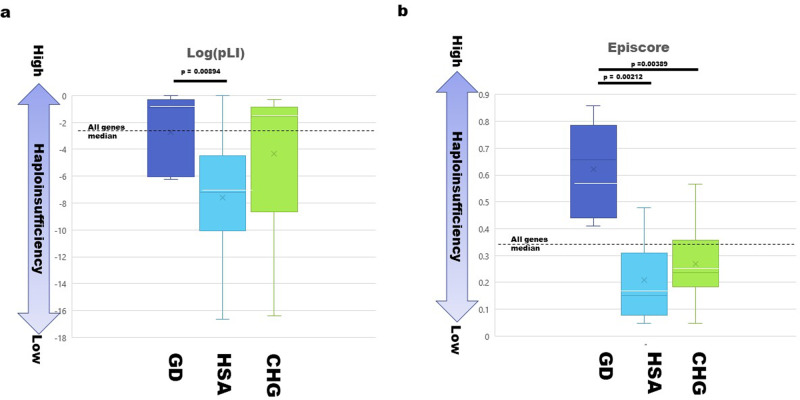
Intolerance of haploinsufficiency in the three gene categories. Categorical box-whisker plots of log of pLI (**a**) and Episcore (**b**) for gonadal dysfunction (GD), disorders in hormone synthesis or action (HSA), and central causes of hypogonadism (CHG). The x-axis indicates DSD-causative gene categories. The y-axis indicates the log of pLI and Episcore for **a** and **b**, respectively. The upper and lower ends of boxes indicate 25% and 75% of each category. Horizontal white lines in the boxes indicate parameter medians. Whiskers in the boxes indicate the minimum and maximum values of each category. Statistically significant differences (Student’s *t* test) are indicated at the top of the plots. Median values for each parameter are indicated using broken horizontal lines.

The distributions of Log(pLI) and Episcore for the three categories of DSD genes are presented in Fig. 3a and b. GD genes displayed lower intolerance to haploinsufficiency, whereas HSA genes showed relatively high tolerance to haploinsufficiency in both parameters. Interestingly, the CHG category adopted a distinct pattern: the log (pLI) was similar to that of GD (Fig. 3a), but Episcore was similar to that of HSA (Fig. 3b). These results suggest that combining these two parameters will be useful for estimating haploinsufficiency.

## Discussion

We selected 32 recessive DSD- and CHG-related genes, including 31 autosomal recessive genes and *AR* on the X chromosome, and then categorized them into three (GD, HSA, and CHG) groups. We classified the variants of the 32 genes in 8.3KJPN using ClinVar, InterVar, HGMD, and MAFs and evaluated the degree of pathogenic significance.

Twenty-eight candidate variants with novel and reliable pathological significance were identified in the Japanese general population. When the panel diagnosis of DSD becomes widespread in Japan, different variants will be identified, and their pathological significance will be discussed. The information on the novel variant candidates identified in this study may be useful.

The carrier frequencies of the recessive DSD- and CHG-related genes were estimated as follows: nearly one in 157 (Set 1), one in 73 (Set 2), one in 40 (Set 3), and one in 16 (Set 4) individuals. However, these data alone do not help us to determine which set of estimated carrier frequencies approximates the real situation. By investigating biallelic inactivation (BI) of the genes, we can evaluate which set most accurately approximates the true carrier frequencies. The investigation of BI suggests the possibility of false positives among the HGMD DM variants. We suggest that the most reliable and conservative estimation was for Set 1 and that Set 3 may better represent reality.

In the simple autosomal recessive form of genetic disease, individuals in the homozygous or compound heterozygous state of pathogenic variants constitute symptomatic cases. However,　since most variants of DSD-causative genes result in a disease phenotype in a sex-dependent form, individuals in the homozygous or compound heterozygous state for pathogenic variants do not necessarily exhibit phenotypes. Thus, investigation of BI for DSD-related genes with autosomal recessive inheritance cannot estimate the true frequency of DSD. However, it is very meaningful that we were able to validate our inclusion criteria from the BI investigation.

Most of the LOF variants were found in HSA genes but not in GD genes. Two parameters for haploinsufficiency of a gene (pLI and Episcore) were compared among the three categories, with GD exhibiting the highest intolerance to haploinsufficiency. Conversely, GD genes were more intolerant to haploinsufficiency. The medians of two parameters for intolerance to haploinsufficiency (pLI and Episcore) were significantly higher for GD than the other two categories (HSA and CHG), as well as in the whole set of human genes analyzed. This might be associated with the paucity of LOF variants in the GD category, even though we selected recessive causative genes. Homozygosity or compound heterozygosity of hypomorphic alleles of GD genes might cause severe phenotypes. Some modifiers may alleviate hypomorphic phenotypes in carriers of GD category variants. The three gene categories had distinct patterns of LOF variants; the high-fidelity genomic data in 8.3KJPN indicated good concordance with the biological effects of DSD- and CHG-related genes.

Our study has several limitations. Some valid pathogenic variants in DSD-causative genes in the Japanese population might have been missed. The 8.3KJPN database and its earlier version 3.7KJPN^35^ were constructed based on the GATK Best Practice workflow^35^. To maintain the integrity of the participants’ records in the reference panel, we excluded genomic data showing discordance with the donors’ reported sex from 8.3KJPN. Therefore, the genome reference panel did not include pathogenic variants from DSD patients among the prospective cohort participants. We could not include the genes on the Y chromosome in our study because 8.3KJPN does not include Y variants. The *SRY* gene is a critical inducer of the male phenotype, and we previously identified mutations in it in three XY patients with pure gonadal dysgenesis^36^. These limitations might be overcome through whole-genome sequencing and further Y chromosome data from Tohoku Medical Megabank cohort participants.

This study reconfirmed the difficulty of analyzing the *CYP21A2* gene by NGS processing. The *CYP21A2* pseudogene *CYP21AP1* contributes to misalignment of the *CYP21A2* region during NGS processing, causing errors^37^. Therefore, the utility of the MLPA method in conjunction with nested PCR has been widely recognized for genetic analysis of the *CYP21A2* gene^38^.

This is the first study to investigate autosomal recessive variants of DSD and CHG in the general population of Japan. We focused on the recessive form of genes because of the population size and background using the 8.3 KJPN and determined the types and frequencies of variants. These results will be very useful for genetic diagnosis and genetic counseling in cases of DSD and CHG, especially when prioritizing target genes based on MAF to identify genes responsible for the phenotype of a patient.

## Supplementary information

Supplementary table 1

Supplementary table 2

Supplementary table 3

Supplementary table 4

Supplementary table 5
