# Supplementary material for: Pathological variants in genes associated with disorders of sex development and central causes of hypogonadism in a whole-genome reference panel of 8380 Japanese individuals
Source: Hum Genome Var. 2022 Sep 28;9:34. doi: 10.1038/s41439-022-00213-w (PMC9519586; doi:10.1038/s41439-022-00213-w)
Supplement: Supplementary file 3 — Supplementary table 3 [file 41439_2022_213_MOESM3_ESM.pdf]

**Supplementary Table 3**. ClinVar P variants in DSD and CHG causative genes of 8.3KJPN

| Gene Symbol | chr | pos | ID | REF | ALT | QUAL | FILTER | AC | AN | amino acid change | CLNSIG | Platform bias test | Platform bias | AF | gnomAD AFR | gnomAD AMR | gnomAD ASJ | gnomAD EAS | gnomAD NFE |
|---|---|---|---|---|---|---|---|---|---|---|---|---|---|---|---|---|---|---|---|
| CYP21A2 | 6 | 32006291 | rs9378251 | C | T | . | VQSRTrancheSNP99.80to99.90 | AC=10 | AN=16760 | p.Pro31Leu | CLNSIG=Pathogenic | TOMMO_PLATFORM_BIAS_TEST_PVALUE=0.006 | . | AF=0.0006 | 0.001 | . | . | . | 0.0008 |
| CYP21A2 | 6 | 32006909 | rs387906510 | GGAGACTAC | G | . | VQSRTrancheINDEL99.00to99.90 | AC=2 | AN=16760 | p.Gly111fs | CLNSIG=Pathogenic | TOMMO_PLATFORM_BIAS_TEST_PVALUE=1 | . | AF=0.0001 | . | . | . | . | 0.0001 |
| CYP21A2 | 6 | 32007203 | rs6475 | T | A | . | VQSRTrancheSNP99.60to99.80 | AC=16 | AN=16758 | p.Ile173Asn | CLNSIG=Pathogenic | TOMMO_PLATFORM_BIAS_TEST_PVALUE=0.9735 | . | AF=0.001 | 0.0007 | 0.0036 | . | 0.0013 | 0.001 |
| CYP21A2 | 6 | 32007593 | rs6476 | T | A | . | VQSRTrancheSNP99.60to99.80 | AC=3 | AN=16760 | p.Met240Lys | Pathogenic* | TOMMO_PLATFORM_BIAS_TEST_PVALUE=1 | . | AF=0.0002 | 0.0184 | 0.0024 | . | . | 0.0001 |
| CYP21A2 | 6 | 32007887 | rs6471 | G | T | . | VQSRTrancheSNP99.80to99.90 | AC=22 | AN=16760 | p.Val282Leu | CLNSIG=Pathogenic | TOMMO_PLATFORM_BIAS_TEST_PVALUE=0.5512 | . | AF=0.0013 | 0.0046 | 0.0253 | 0.063 | 0.0032 | 0.0081 |
| CYP21A2 | 6 | 32007959 | rs267606756 | G | GT | . | VQSRTrancheINDEL99.00to99.90 | AC=15 | AN=16760 | p.Leu308fs | CLNSIG=Pathogenic | TOMMO_PLATFORM_BIAS_TEST_PVALUE=0.002 | . | AF=0.0009 | . | . | . | 0.0006 | 0.0001 |
| CYP21A2 | 6 | 32008198 | rs7755898 | C | T | . | VQSRTrancheSNP99.80to99.90 | AC=216 | AN=16756 | p.Gln319* | CLNSIG=Pathogenic | TOMMO_PLATFORM_BIAS_TEST_PVALUE=0.0005 | TOMMO_POSSIBLE_PLATFORM_BIAS | AF=0.0129 | 0.003 | 0.0025 | 0.0145 | 0.0021 | 0.0024 |
| CYP21A2 | 6 | 32008312 | rs7769409 | C | T | . | VQSRTrancheSNP99.90to99.95 | AC=358 | AN=16756 | p.Arg357Trp | CLNSIG=Pathogenic | TOMMO_PLATFORM_BIAS_TEST_PVALUE=0.0005 | TOMMO_POSSIBLE_PLATFORM_BIAS | AF=0.0214 | . | . | . | 0.0013 | . |
| CYP21A2 | 6 | 32008874 | rs200005406 | G | A | . | VQSRTrancheSNP99.60to99.80 | AC=3 | AN=16760 | p.Arg484Gln | CLNSIG=Pathogenic | TOMMO_PLATFORM_BIAS_TEST_PVALUE=1 | . | AF=0.0002 | . | . | . | . | . |
| STAR | 8 | 38001877 | rs104894085 | G | A | . | VQSRTrancheSNP99.60to99.80 | AC=11 | AN=16760 | p.Gln258* | CLNSIG=Pathogenic | TOMMO_PLATFORM_BIAS_TEST_PVALUE=0.8516 | . | AF=0.0007 | . | . | . | . | . |
| AKR1C2 | 10 | 5037962 | rs13222 | A | G | . | VQSRTrancheSNP99.80to99.90 | AC=8 | AN=16758 | NA (upstream) | CLNSIG=Pathogenic | TOMMO_PLATFORM_BIAS_TEST_PVALUE=0.0145 | . | AF=0.0005 | 0.0602 | 0.005 | 0.0079 | 0.0007 | 0.0016 |

*No assertion criteria provided.
