# Supplementary material for: Pathological variants in genes associated with disorders of sex development and central causes of hypogonadism in a whole-genome reference panel of 8380 Japanese individuals
Source: Hum Genome Var. 2022 Sep 28;9:34. doi: 10.1038/s41439-022-00213-w (PMC9519586; doi:10.1038/s41439-022-00213-w)
Supplement: Supplementary file 4 — Supplementary table 4 [file 41439_2022_213_MOESM4_ESM.docx]

| Supplementary Table 4. Distribution of candidate loss-of-function variantsin DSD- and CHG-associated genes | | | | | | | |
| --- | --- | --- | --- | --- | --- | --- | --- |
|  |  | Functional annotations | | | | |  |
|  |  |  |  | Loss-of-function (questionable) | | | |
| Category | Gene | Non-synonymous | In-frame deletion | Stop-gain | Splicing | Frameshift | Total LOF |
| GD | 6 | 2 | 0 | 1 (1) | 0 | 0 | 1 (1) |
| HSA | 19 | 50 | 2 | 7 | 7 (1) | 10 | 24 (1) |
| CHG | 8 | 5 | 0 | 4 (2) | 0 | 3 | 7 (2) |
| Total | 33 | 57 | 2 | 12 (3) | 7 (1) | 13 | 33 (4) |
